# A Course of Lectures on Dental Physiology and Surgery, Delivered at the Middlesex Hospital School

**Published:** 1848-04

**Authors:** John Tomes

**Affiliations:** Surgeon Dentist to the Hospital.


THE AMERICAN

### Journal of Dental Science.

Vol. VIII.] APRIL, 1848. [No. 3.

### ARTICLE I.

*A Course of Lectures on Dental Physiology and Surgery, delivered at the Middlesex Hospital School.* By JOHN TOMES, ESQ., Surgeon Dentist to the Hospital.

(Continued from p. 147.)

### LECTURE X.

**DENTAL CARIES—PHYSICAL CHANGES OF THE DENTINE AND ENAMEL—CAUSE OF CARIES—THEORIES OF DENTAL CARIES—ANALOGY OF DENTAL TO OSSEOUS CARIES—TABLES OF THE RELATIVE FREQUENCY OF CARIES IN THE DIFFERENT TEETH, AND IN DIFFERENT PARTS OF THE SAME TEETH—CAUSES AND THE TREATMENT OF CARIES.**

### *Diseases of the Dental Tissues.*

CARIES, dental gangrene, or decay, are the various names by which the disease I am about to describe is known. It is but of little consequence which of these we select for our present use; but as caries is the oldest, as is as expressive of the nature of the disease as the others, while it has the advantage of being short, we will, if you please, adopt that term.

Caries may be defined to be, death and subsequent progressive decomposition of a part or the whole of a tooth.

The physical changes which mark the progress of the disease are first observable on the periphery of the dentine, under the enamel, which latter is, over the affected spot, either itself slightly discolored, or unusually opaque and white. The diseased dentine generally becomes changed in color, and assumes a brown hue, more or less deep according to the rapidity with which the decay has advanced. The slower the process of decomposition the deeper will be the color of the carious portion, and *vice versa.* The decomposed condition advances gradually from the surface towards the centre of the tooth, invariably taking the course of the dental tubes; and as the various branches of these converge, in their passage inwards, to join the main tubes, which open on the surface of the pulp cavity, the diseased mass is consequently of conical form.

After a while the surface of the dentine immediately under the enamel becomes softened by the loss of its earthy ingredients; the enamel itself affected, and no longer supported by the subjacent dentine, falls in, and a cavity is formed in the tooth, which increases in depth, following the course of the tubes, and at the same time extends laterally by the extension of the disease on the surface of the dentine, and from thence inwards. By a continuance of this destructive process an opening is formed into the pulp cavity, and, eventually, the whole of the crown of the tooth is destroyed. After the removal of the earthy constituents the gelatine of the tooth is at first firm and elastic, like cartilage, but it soon softens by decomposition, becomes disintegrated, and is lost in the saliva: thus a cavity is formed in the tooth, which is occupied by the fluids of the mouth and small particles of food. The crown being destroyed, the disease is frequently arrested. The roots of the affected tooth having a higher degree of vitality than the more exposed crown are proof against the influence which lead to the destruction of the latter. You will meet with a few instances where the decay has been completely arrested after destroying only a portion of the crown. In these cases the surface of the so-formed cavity will be found of a dark brown color, but hard and highly polished, like the surface of eburnated bone.

The fissures in the masticating surface of the grinding teeth, or those surfaces of the teeth which lie in contact with the contiguous teeth, are the situations usually attacked by caries.

The diseased action is not attended by severe pain, but I am disposed to think that even early in the disease there is in almost all cases a feeling of slight uneasiness in the affected tooth, though the degree may be so slight as to escape detection at the time, or lead only to the supposition that some foreign matter has got between the teeth. I am induced to adopt this opinion, though at variance with several authors of distinction, both from the description given by patients who have suffered from caries, and also from personal experience in this unpleasant malady. On several occasions my attention has been drawn to a tooth by a sensation not, perhaps, amounting to pain, but to slight discomfort only, which has lasted for a few minutes. After a recurrence, from time to time, of this unpleasant sensation, I have examined the tooth, and have invariably found that caries has commenced in the situation from whence the uneasiness seemed to proceed.

Again, a few cases are met with in which there is considerable pain attending the progress of caries, long before the pulp is at all affected by exposure, or by the contact of partially decomposed dentine. The tooth is, from the commencement, highly sensitive: the contact of hot or cold fluids induces severe pain, and any attempt to remove the carious dentine, though the disease has existed but for a short time, and is very slight in extent and depth, produces such unbearable pain, that the attempt is for the time obliged to be abandoned.

The causes and nature of caries are variably estimated by different writers, but the theories advanced, in explanation of the subject, may be arranged under one of the two following heads. The first includes those in which caries is in itself considered a vital action and the result of inflammation; the second includes those in which caries is regarded wholly as the result of chemical action, and caused by the presence of decomposing matter lodged in the interstices of the enamel. I should, however, at starting, state that these doctrines were promulgated long before the structural anatomy of the teeth was understood. Fox endeavors to show that dental is in nature similar to osseous caries; he assumes that separation of the periosteum is the cause of caries in the bones, and that separation of the pulp from the surface of the pulp cavity is the cause of dental caries: and, further, that in each case the separation is the result of inflammation. Mr. Bell advances the opinion, that caries is the result of inflammation in the dentine itself, and that, from the low degree of organization of that tissue, it is unable to recover from active inflammation. He says, "The true proximate cause of dental gangrene is inflammation, and the following appears to me to be the manner in which it takes place. When, from cold, or any other cause, a tooth becomes inflamed, the part which suffers most severely is unable, from its possessing comparatively but a small degree of vital power, to recover from the effects of inflammation, and mortification of that part is the consequence. Many subsequent writers have, with slight and unimportant variations, supported the views of these great authorities on dental surgery. Mr. Robertson, of Birmingham, stands pre-eminent amongst those who assume that caries is nothing more than chemical decomposition of the tooth, and who assert that the tissues composing the teeth are devoid of life, and that, therefore, vital action cannot occur in them.

These two classes of opinions are sufficiently at variance with each other. I think, however, I shall be able to convince you that they are, taken together, both right, but that neither, taken alone, will account for all the phenomena of caries.

I believe that the dentine from abnormal action loses its vitality, and with the loss of vitality the power of resisting chemical action, and that, consequently, the dead part is under favoring circumstances decomposed by the fluids of the mouth. Further, I conceive that the causes producing the abnormal action may have been applied locally to the tooth itself, or may have had a constitutional origin, and, therefore, have acted through the nerves or the circulating fluids.

To support these views it will require that I should go a little more at length into the subject. Time, however, cannot be misspent when devoted to the consideration of a disease so universal in its presence, and at the same time so prejudicial to the health and comfort of those whose teeth are affected. You will recollect that the surface of the dentine is composed of the terminal branches of the tubes, and that, in addition to these, there are a vast number of small cells present in the part which is immediately covered by the enamel. With these cells the tubes communicate, and in addition some few pass into the enamel. On the surface of the dentine so composed the first physical changes denoting the existence of caries are observable.

I have shown you that a dead tooth is pervious to fluids. A tooth that has been some little time removed from the mouth, and kept in a dry situation, becomes perfectly opaque, especially the enamel, and the more so if the tooth be taken from the young subject. But if we immerse the dry, and consequently opaque tooth in water, it will absorb gradually through the external surface a considerable quantity of the fluid, and thereby become slightly translucent. From these circumstances we learn that the fluids of the mouth may permeate the substance of a tooth if there is no vital action to oppose their doing so, and supposing them to have permeated, they may, if they be of an active character, and there be present in the dentine no restraining power, decompose the substance of the tooth. As the solvent menstruum enters from without, the more external part of the dentine will be the first to be affected; again, the fluid finding a ready passage through the tubes, travels inwards towards the pulp cavity, contracting in its extent as the tubes converge. Further, we find decay travels under the enamel on the surface of the dentine: this circumstance is fully accounted for by the presence of the numerous cells and their frequent connection with each other in this part. It will be inquired why, as the decomposing agent enters through the enamel, is it that that tissue is not the first to suffer? Strictly speaking it may be the first, but as a much stronger solvent is required, or the longer application of the same solvent, to produce similar impressions on the enamel, the effect is first perceived in the dentine. If we place a tooth in a weak solution of muriatic acid we shall find that the exposed dentine is affected before the enamel, and the exposed dentine before that covered by enamel. As a further testimony of the correctness of this view of the chemical solution of the earthy ingredients of dead dentine in caries, it may be urged that the action first shows itself in a situation where a fluid or semifluid might be kept in undisturbed contact with the surface of the tooth for some time: thus in the fissures of the masticating surface of the molares, or on the laterally opposed surfaces of the teeth, in either of which situations the debris of food may remain unmolested for hours, and thus enable the fluid with which it is saturated to enter the dentine. If an acid be the solvent, removing the earth of teeth, it would seem probable that its presence might be detected. With this view I have repeatedly applied litmus paper to carious teeth, both before and immediately after their removal from the mouth, and have almost invariably found strong evidences of the presence of a free acid.

So far we can understand the course which dental caries takes from our knowledge of dental structure, and can understand how the decomposition of the dentine is effected; but this point will be even more intelligible after an examination of the affected tissue with the microscope. A transverse section of carious dentine, rendered soft like cartilage from the loss of its lime, presents a cribriform appearance. The tubuli seem enlarged and rather irregular, quite unlike the figure they present in healthy dentine: this would indicate that the solvent enters and acts upon the parietes of the tubes previous to affecting the inter-tubular tissue, and that the parietes of the tubes are, therefore, the first to disappear. I feel quite certain that in the cases I have examined, and they are numerous, that the parietes of the tubuli so distinguishable in healthy dentine have almost, if not wholly, disappeared with the removal of the lime.

As regards the source of the solvent agent some little inquiry is needed. The saliva is in many cases, where decay is rapidly progressing in several teeth at the same time, strongly acid. The stomach frequently during the day secretes gastric juice, which, if eructated in a very small quantity, would be sufficient to keep up the dental decomposition already commenced. In seeking the source of the acid you must not forget that the actual amount of phosphate of lime contained in a tooth is small; and, further, that the destructive action is often very slow and spreads over many months. The presence of the least appreciable quantity of solvent would, therefore, be required, and even this where the decay is slow only at intervals. You will find a statement in Simon's Animal Chemistry (translated by the Sydenham Society,) to the effect that the saliva under certain circumstances is acid, and that this condition is not uncommon after salivation. But quite apart from the saliva as a source of acid, I think we take a sufficient amount in our food to effect the slow decomposition of dead dentine.

It is not rare to meet with a case in which the saliva is strongly acid, either in itself or from the admixture of fluids from the stomach; and, should the gums have receded and left the necks of the teeth unprotected from the direct contact of the saliva, the exposed portion below the enamel will be deprived of its earthy matter, and the crown will be at last either cut off from the fang by the decomposition of the neck, or will be itself in a great part decomposed. We commonly see instances of this in patients who from indisposition take some of the mineral acids.

Artificial teeth are frequently made of natural human teeth, fixed on a plate fitted to the gums. In these, decomposition goes on just as in the natural teeth; but there is this difference; you may always know where decomposition will take place, namely, wherever the dentine is unprotected from the contact of the oral fluids; and in most persons, though not in all, the artificial teeth will, in course of time be wholly decomposed. Thus, in the bicuspides, the decay will commence on the masticating surface, if the pin fixing it to the plate be brought through and insufficiently rivetted: also, on the surface fitted to the plate. In either of these situations the saliva may get in and remain in contact with the surface of the dentine.

Thus, we have ample proof that dead dental tissue will, under favorable circumstances, be gradually decomposed by the fluids of the mouth; but there are conditions which, if present, entirely prevent or greatly retard its decomposition. The saliva may be so charged with phosphate of lime that it is thrown down in considerable quantity wherever the salivary fluid remains at rest. It collects in large masses upon the posterior part of the necks of the lower incisores; and I have seen the cavities of decayed teeth completely filled with the deposit.

Supposing, then, this condition to prevail, the oral fluids, that is, the saliva and mucous secretion, being already saturated cannot dissolve more phosphate of lime; and dental decomposition, therefore, cannot commence, or, if commenced, cannot advance. It must not, however, be supposed from what I have said that in all cases where phosphate of lime (or tartar, as it is more commonly called) is present on the teeth that dental decay will be arrested; the state of the saliva may have changed since the deposit of the tartar, or, though the saliva may be alkaline, the secretion from the mucous membrane of the gums may be acid; and, supposing such to be the case, we should hardly expect that the secretion thrown out from the membrane between the teeth would be neutralised, as mucus does not readily mix unless agitated with another fluid; and such agitation would not take place in that lodged between the teeth, or in fissures or cavities of teeth.

Dead dentine will also resist the action of the oral fluids, if the surfaces be highly polished. Thus, if the surfaces of artificial teeth constructed wholly of dentine be highly polished, and the polish be constantly kept up by the wearer, the teeth will not be acted upon, unless the decomposing agent be unusually strong; but, if they are polished in one part, and left rough from the file in another, decomposition will quickly commence on the rough, while it is resisted by the polished surface. You will scarcely find a set of artificial teeth that have been worn in which this point is not more or less illustrated. Those surfaces which are exposed to the action of the tongue, or of the springs, are kept highly polished, and in these situations decay is effectually resisted, while the parts lying in contact with the gums are gradually softened, and the teeth become unfit for use. Again, if a tooth from any cause (as by filling) be deprived of enamel, the exposed dentine will in most cases soon be robbed of the lime, unless the surface be polished and kept in that condition. We have frequent opportunities of observing exposed and eburnated dentine in teeth that have been worn by the action of the antagonist teeth, and these, we see, have resisted decomposition.

From the foregoing facts, we are led to conclude that dead dental tissue, when retained in the mouth will be decomposed or not, in accordance with the circumstances under which it is placed, and that the decomposition is effected by agents applied externally.

Before leaving this part of the subject, I will call your attention to a very curious fact in regard to bone. Mr. Gulliver, by a series of experiments instituted to ascertain whether dead bone was absorbed or not, found that a portion of human bone introduced into the medullary cavity of a rabbit's tibia not only remained there without exciting inflammation, but became united to the adjoining bone; and in other experiments, where union was not effected, living bone was deposited upon the dead bone.*

It is thus sufficiently apparent that dead bone may exist without our knowledge of its presence.

I find there is some difficulty in making an accurate comparison between the caries of teeth and of bones. Surgical writers are not very definite upon the point as to how the osseous tissue is affected; neither can I find any good account of the chemical nature of the discharge from a carious bone.

* Medico-Chirurgical Transactions, vol. xxi, p. 16, expt. 16.—A portion of the shaft of the human tibia was weighed and introduced into the tube of a rabbit's tibia; seven weeks after which the animal was killed. The limb was macerated for three months during the summer, when a part of the circumference of the tibia being removed to expose the foreign bone, it was found firmly adherent to the inner surface of the rabbit's tibia, and the union was effected by true osseous substance, as proved by the analysis of Dr. Davy, E. P. B., 56, in the museum of the Army Medical Department.

In South's translation of Chelius, I find, in a note from Miescher, under the article *caries*, the following statement:—"We see it asserted in the works of not a few writers, that in a carious ulcer the pus may be perceived to be, as it were, sandy to the touch, and containing *bony particles* oftentimes pretty apparent." Again, he says, "the whole surface, therefore, attacked with caries presented various stages of separation of the dead plates, by which the roughness felt with the probe was easily explained, as well as the corroded appearance of *the softened bone*, which, though less conspicuous, is discovered after the separation of the large piece of bone." Now the only means by which a piece of dead bone could be softened would be by chemical decomposition, the process being similar to that engaged in the destruction of dentine, thereby establishing a strong similarity between the caries of bone and of teeth. In caries, a portion of bone loses its vitality: the neighboring bone becomes inflamed, as do the surrounding soft textures. This leads to the formation of an abscess, which opens on the surface of the body, and also to the separation of the dead bone from the living, by the absorption of a thin layer of the living which lay in immediate contact with and connected the dead to the living bone; the former may or may not have been decomposed, the condition depending upon the nature of the fluids with which it has been bathed.

In dental caries, a portion of dentine loses its vitality, but does not, like dead bone, become separated from the living, which difference may be attributed to the very different circumstances under which it is placed, and also to its lower degree of vitality; but, on the other hand, the dead dentine will, like dead bone, be decomposed by solvent fluids, when such are present.

The crown of a tooth, the more external tissues of which are dead, the pulp and internal dentine being living, I conceive to be exactly similar in condition to an eburnated bone. The synovia of the joint in contact with the bone is not of a nature to decompose the bone, so no chemical change takes place in the osseous tissue, which, over the exposed surface of the bone, is by friction highly polished.

The saliva bathing the dead surface of tooth may not contain active ingredients; and in that case the tooth will not be decomposed.

Again, bone may be dead, but there may be no inflammation of the neighboring parts, and the dead will not be separated from the living. This we may infer from Mr. Gulliver's experiments, in which dead was introduced into the medullary cavity of living bone, became perfectly united, and the limb in which the experiment was tried sound and healthy. That state, which is occasional only in bone, is constant in dentine; dead dentine is never separated from the living by the absorption of the connecting layer of the latter.

The analogy in the diseases of the two tissues is further established by the fact that in many cases the presence of caries in the crown of a tooth leads to the deposition of dentine by the pulp opposite the point to which the disease is advancing. The dental deposit may exist only in the form of a few granules of dentine, or may extend to the formation a plate. From what has been said, you will have gathered that it is not at all times easy to know whether any or how much dentine be dead in a tooth; and the same observation may be applied to bone, as, from the foregoing statements, it would appear that all indications of death may be absent. Death of a part by no means implies immediate discoverable physical change. In a dense tissue of naturally low sensibility, we have no means of recognising the loss of vitality, except by the occurrence of physical change in the part. The color must change, decomposition must commence, or there must be alteration of figure, before we become aware of the event. Even in soft tissue, we only recognise death by finding indications of decomposition in the suspected part.

In a tooth, then, you can only pronounce that the crown has wholly or in part died, by observing that discoloration or decomposition is present in that part.

*Causes of Dental Caries.*—Many of the causes which favor the chemical decomposition of dental tissue may lead also to the loss of vitality which renders it susceptible of decomposition. Thus, an imperfect development of the enamel on the sides or in the natural fissures of the teeth, and a consequent exposure to the dentine to the contact of whatever may be taken into the mouth, is a very frequent cause of caries. There are but few who have not at some time experienced most unpleasant sensations, oftentimes amounting to pain, in teeth perfectly sound, when acids or sweets are taken into the mouth. In these cases the irritating fluid must have passed through the enamel and come in contact with the dentine, which we know is sensible, while the enamel is not.

Patients are occasionally met with, who state that they were attacked with pain in several sound teeth; that the pain left, but after the lapse of a short time, the teeth decayed, and left only stumps.

After fever, and especially scarlet fever, teeth, before quite sound, in some cases rapidly decay. The same occasionally occurs after salivation. Children of a scrofulous diathesis are liable to lose their teeth by caries at an early period; also, persons who suffer from indigestion with excess of gastric acid. A residence in a low damp situation is said to be a frequent cause of this disease.

Many writers assume that where teeth are crowded, great pressure is exerted upon the lateral surfaces in contact, that this causes a disarrangement of the fibres of the enamel, and thus leads to disease. I am disposed to doubt whether this is a frequent cause; you will very commonly see a most crowded set of teeth, and yet no caries. Again, if lateral pressure were a frequent cause, you would naturally expect those teeth the most subject to pressure, the first to decay, and, further, the parts of the tooth subject to the pressure would be those first affected. Yet neither the one nor the other point is borne out by experience; on the contrary, the first permanent molar is the most liable to become diseased, and the masticating surface, the point most commonly attacked, and yet this tooth is less liable during its development and eruption to lateral pressure than any other in the mouth.

*Relative Liability of the Various Teeth to Caries.*—For some time past I have kept a register of every case that has come under my treatment at the hospital. The case-book has a tabular arrangement, and in each entry the name and age of the patient, and the teeth affected, are noted each in their proper columns. In those cases requiring the removal of teeth (and forty-nine out of fifty are of this character) the situation in which disease has commenced, and the instrument used in extracting the tooth, are also noted. The cases relating to permanent teeth which occurred in 1845, and the first six months of 1846, have been partially summed up, and the results arranged in the forms I now lay before you. In those instances in which it has been difficult to tell with certainty, from the extent of destruction, where the caries commenced, the probable situation has then been stated. Excepting this occasional source of error, the tables may be perfectly relied on.

I should state in explanation, that, in each case requiring removal, whatever may have been the condition of the tooth rendering immediate operation necessary, if caries was the cause of that condition, the entry was made under the head of caries. A carious tooth will often lead to alveolar abscess, but when this has been the case, as caries was the primary cause of the disease, the case has been registered as caries.

Again, destruction of the crown by caries will occasionally be followed by exostosis of the fang: the case has then also been registered as caries.

Under the head of looseness are included teeth loose from various causes, though nine-tenths have been from absorption of the alveoli, the teeth themselves being sound.

It would be a waste of time to enter into any description of the relative liability of each part of a tooth to disease, as a perusal of the following tables will give you all the definite information I possess upon the subject, and that, too, in a much more satisfactory form than a mere statement would be.

*Statistical Tables of* 1736 *Cases of Dental Disease, treated at the Middlesex Hospital.*

Of the 1736 permanent teeth extracted from all causes:

44 were central incisores,
65 " lateral incisores,
39 " canines,
150 " first bicuspides,
231 " second bicuspides,
642 " first molares,
389 were second molares,
176 " third molares.
Of these, 1480 were removed for the relief of diseases commencing in caries of dental tissues.

Of the 1787 permanent teeth extracted from all causes:

44 were CENTRAL INCISORES: of these
20 " from males, and
24 from females,
36 from the upper, and
8 from the lower jaw.

Of the 36 from the upper jaw:

13 were stumps,
7 " fractured.
7 " loose,
2 " dead,
1 for decay on the lateral surface,
1 inflammation of gum and periosteum,
3 for abscess,*
2 projected.*

Of the 8 from the lower jaw:

5 were loose from the absorption of the alveoli,
1 was dead,
2 were out of line.

Of the 1736 permanent teeth extracted from all causes:

65 were LATERAL INCISORES: of these
33 were from males, and
32 from females;
51 from the upper, and
14 from the lower jaw.

Of the 51 from the upper jaw:

2 were fractured,
8 for want of space,
7 were loose from absorption of the alveoli,
4 for decay on the posterior surface,
8 " external "
8 " lateral "
1 for decay on the masticating surface,
1 " not noted where,
14 were stumps,
1 for cause not registered,
1 for necrosis,
2 for abscess.

Of the 14 from the lower jaw:

6 were loose from absorption of the alveoli,
6 for want of space,
1 for cause not registered,
1 for tic douloureux.

TABLE IV.—Of the 1736 permanent teeth extracted from all causes:

39 were CANINES: of these
19 " from males, and
20 " from females,
29 were from the upper, and
10 " from the lower jaw.

Of the 29 from the upper jaw:

In 2 cases the lateral surface next the lateral incisores was attacked with caries,
2 cases the lateral surface next the bicuspides,
" lingual surface,
" labial surface,
1 " masticating surface,
1 case the situation not noted,
5 " stumps,
7 " looseness,
9 " out of line,
1 " inflammation of the gum, involving the periosteum of the fang,
1 " cause not registered.

* The teeth sound.

### Of the 10 from the lower jaw:

In cases the lateral surface next the lateral incisores was attacked with caries,
1 " lateral surface next the bicuspides,
1 " lingual surface,
" labial surface,
1 " stumps,

3 cases of looseness producing irritation and pain,*
2 " out of line,*
1 " abscess in the alveolus,*
1 " tic douloureux was supposed to arise from the presence of the tooth.

### TABLE V.—Of the 1736 permanent teeth extracted from all causes:

150 were FIRST BICUSPIDES: of these,
70 " from males, and
80 " from females,

121 were from the upper, and
29 " from the lower jaw.

### Of the 121 from the upper jaw:

In 19 cases the anterior surface was attacked with caries,

45 " posterior surface,

3 " lingual surface,

2 " buccal surface,

2 " masticating,

" situation not noted,

22 " stumps only were present,

9 " looseness occasioning irritation and pain,

13 cases the space was required for the anterior teeth,*

1 " necrosis,*

1 " inflammation of the gum,*

1 " abscess in the alveolus,*

1 " tic douloureux,*

1 " cases not registered,

1 " the tooth was loose, and when removed found to have no fang, none having been developed.

### Of the 29 from the lower jaw:

In 7 cases the anterior surface was attacked with caries,
7 " posterior,
7 " lingual,
2 " buccal,

In 5 cases the stumps only remained,
4 " looseness occasioning irritation and pain,
2 " space was required for the anterior teeth.

### TABLE VI.—Of the 1736 permanent teeth extracted from all causes:

231 were SECOND BICUSPIDES: of these,
115 were from males, and
116 from females,

147 from the upper, and
84 from the lower jaw.

### Of the 147 from the upper jaw:

In 30 cases the anterior surface was attacked with caries,

52 " posterior " "

2 " lingual " "

1 " buccal " "

4 " masticating " "

15 " looseness,

1 " space,

1 " necrosis,

" inflammation,

3 " abscess,

In 1 case the situation not noted,
29 " stumps,
In these 119 cases, caries was the primary cause of the loss of the teeth.

1 case tic douloureux,
1 " fracture,
1 " connected with dead bone,
5 " causes not registered.

### Of the 84 from the lower jaw:

In 16 cases the anterior surface was attacked with caries,
29 " posterior " "
3 " lingual " "
3 " buccal " "

In 5 cases the masticating surface was attacked with caries,
14 " stumps " "
In these 70 caries was the primary cause of the loss of the teeth.

* The teeth apparently sound.

10 cases looseness,
1 " necrosis,

1 case tic douloureux,
2 " for causes not registered.

TABLE VII.—Of the 1736 permanent teeth extracted from all causes:

642 were FIRST MOLARES: of these,
337 were from males, and
305 from females;

259 from the upper, and
383 from the lower jaw.

Of the 259 from the upper jaw:

In 71 cases the anterior surface was attacked with caries,
40 " posterior " "
7 " lingual " "
6 " buccal " "
75 " masticating " "
" situation disease not noted,
35 " stumps only remained.
In these 234 cases, caries was the primary cause of the loss of the teeth.

11 cases looseness, occasioning irritation and pain,
1 " necrosis of the anterior fang produced pain,
2 " inflammation of the gum, teeth sound,
5 " pain in the teeth rendered the removal advisable—teeth sound,
6 " causes not registered.

Of the 383 from the lower jaw:

In 68 cases the anterior surface was attacked with caries,
54 " posterior " "
6 " lingual " "
22 " buccal " "
177 " masticating " "
38 " stumps only remained.
In these 365 cases, caries was the primary cause of the loss of the teeth.

In 5 cases looseness, occasioning irritation and pain,
2 " pain rendered removal requisite,
1 " abscess of the alveolus,
In these 8 cases the teeth were apparently sound.
6 cases causes not registered.

TABLE VIII.—Of the 1736 permanent teeth extracted from all causes:

389 were SECOND MOLARES: of these,
195 were from males, and
194 from females;

141 from the upper, and
248 from the lower jaw.

Of the 141 from the upper jaw:

In 26 cases the anterior surface was attacked with caries,
21 " posterior " "
6 " lingual " "
12 " buccal " "
25 " masticating " "
24 " stumps only remained.
Of these 114 cases caries was the primary cause of the loss of the teeth.

19 cases the looseness occasioning irritation and pain,
1 " necrosis,
1 " abscess,
1 " pain,
2 " inflammation of gums, &c.
1 " fracture of the jaw.
In these 25 cases the teeth were apparently sound.
2 cases causes not registered.

Of the 248 from the lower jaw:

In 22 cases the anterior surface was attacked with caries,
22 " posterior " "
3 " lingual " "
45 " buccal " "
107 " masticating " "
32 " stumps only remained.
Of these 231 cases caries was the primary cause of the loss of the teeth.

9 cases the looseness occasioning irritation and pain,
1 " necrosis,
1 " pain,
In these 11 cases the teeth were apparently sound.
6 cases causes not registered.

TABLE IX.—Of the 1734 permanent teeth extracted from all causes:

176 were THIRD MOLARES: of these,
97 were from males, and
79 from females;

68 from the upper, and
116 from the lower jaw.

Of the 60 from the upper jaw:

| | | | | |
|---|---|---|---|---|
| In 12 cases, | the | anterior surface was attacked with caries, | | |
| 3 | " | posterior | " | " |
| 1 | " | lingual | " | " |
| 12 | " | buccal | " | " |
| 10 | " | masticating | " | " |
| 11 | " | stumps only remained. | | |

In these 49 cases caries was the primary cause of the loss of the teeth,

6 cases looseness, occasioning irritation and pain,
1 " abscess of the alveolus,
1 " inflammation of the gum, &c.
2 " pain without decay.
In these 10 cases the teeth were apparently sound.
1 case cause not registered.

Of the 116 from the lower jaw:

| | | | | |
|---|---|---|---|---|
| In 14 cases | the | anterior surface was attacked with caries, | | |
| 6 | " | posterior | " | " |
| 1 | " | lingual | " | " |
| 21 | " | buccal | " | " |
| 40 | " | masticating | " | " |
| 11 | " | stumps only retained. | | |

In these 93 cases caries was the primary cause of the loss of the teeth.

12 cases looseness, occasioning irritation and pain,
1 " exostosis of the fangs and thickening of the jaw,
1 " the tooth decayed, and producing *tic douloureux*,
1 " abscess of alveoli; tooth sound,
3 " the teeth were developed with crown projecting forward, and producing great pain,
5 " causes not registered.

The consequences of caries are various: the disease may commence and progressively destroy the crown of the tooth, and very little pain may be felt during the process; or, it may be arrested after the destruction of the crown, and the roots of the tooth may remain in the gums, and give little or no inconvenience. But, on the other hand, pain may commence with the disease; the pulp, as the disease advances, may become inflamed, and if the tooth is not removed, the inflammation extends to the dental periosteum, and be followed by alveolar abscess: the pain all the while increasing. Not only alveolar abscess may arise as a consequence of caries, but periostitis of, and subsequent necrosis of, a large portion of the jaw. There is an out-patient attending the hospital at this time who is suffering from necrosis of the right side of the lower jaw, which was occasioned by inflammation set up by a carious tooth. Tumors of the gums may arise as a consequence of caries, as also may fungous growth of the pulp; but, as these diseases will come under our notice at a future lecture, I need not do more than refer to them now.

*Treatment of Dental Caries.*—Lost vitality in a tissue we cannot restore, neither should we gain much if we could, when the loss is confined to that portion of a crown of a tooth protected by enamel, seeing that the dead part is as useful as before, supposing that it be enabled to resist decomposition. In our treatment, then, what we have to do is to enable the affected tooth to resist future chemical change, or, if partially decomposed, to replace the lost part by some substance that will itself resist the action of the saliva, and prevent it from coming in contact with and injuring the dentine; or, if the disease has destroyed the crown of a front tooth, to replace it, if required, and the root be healthy, by an operation called pivoting.

But, as the treatment will to a considerable extent be regulated by the extent and situation of the disease, it will be expedient to describe the treatment adapted to these several conditions.

*Treatment of Superficial Caries.*—If, then, the disease is of very small extent, and indicated by slight discoloration of the enamel, and if, on examination, by a pointed steel probe it is found to be superficial, it will be sufficient, whatever the situation, to remove the affected part by a scaulper, or file, and afterwards to well polish the exposed dentine; at the same time directing your patient to keep the surface polished, otherwise the exposed tissue will soon be decomposed by the saliva should it chance to be acid. It is no uncommon thing to find that many teeth are attacked at the same time; on inquiry, you will generally hear that your patient has acid saliva from indigestion, or has been taking medicine containing a mineral acid. In either case it will be well for the patient to use an alkaline dentifrice, and in the latter to rinse the mouth with a weak solution of carbonate of soda after each dose of acid medicine.

On attempting to remove the carious portion, however superficial and small in extent it may be, the tooth will sometimes be so tender that the operation cannot be borne: the slightest touch of the instrument is attended with intolerale pain. Whenever this is the case, the operation should be postponed till the sensitiveness of the affected part is reduced. The most ready means of effecting this, is by rubbing powdered nitrate of silver, or any other escharotic, on the sensitive part, with the end of a piece of whalebone, or cane. Chloride of zinc is, perhaps, the best application, as the effect is speedy, and the tooth will not be discolored. The operation may then be proceeded with, and should the surface again become tender before the completion, the escharotic must again be applied. A prolonged application of camphorated spirits of wine will sometimes subdue the sensitiveness; but when the disease has attacked the exposed neck of the tooth, commencing on the surface of the cementum, a situation in which we find extreme tenderness more frequently than in any other, the camphorated spirits is difficult of continued application.

But if, instead of finding the disease superficial on examination, the probe sinks to some little depth into the tooth, indicating thereby that to that depth the dentine is deprived of its earthy ingredients, then we must have recourse to the operation of plugging. The softened dentine must be removed by conveniently shaped steel instruments. The whole of the softened tissue having been removed, the cavity must be wiped dry, and then carefully filled with some substance which will resist the action of the constituents of the saliva, and will be sufficiently hard to resist injury in mastication. Metals are the only substances found to combine these qualities. Gold, platina, tin, or lead, reduced to thin foil, and packed tightly in the cavity, forms an efficient plug: gold being the most valuable, the others following in the order in which they are placed. An amalgam of palladium or silver may be used with advantage in cases where the cavity is so situated that it becomes inconvenient to pack densely gold or other foil.

A plug is effective only so long as it perfectly excludes all extraneous matter from the cavity; thence it follows, that in order to fill a cavity with metal foil, considerable compressive force must be used. You will at once, therefore, perceive that should the caries have extended so far as to reach the pulp cavity; though by careful management the softened dentine might be removed; yet the patient would be quite unable to bear pressure unless considerable precaution be used, even in the most favorable case; and when the surface of pulp exposed is considerable, the operation could not be borne, and if borne, would produce inflammation of the pulp. In many cases, however, your patient will not apply for relief until the disease has not only progressed to the pulp cavity, but the softened dentine removed so as to make a communication between the pulp cavity and the mouth, thus exposing the sensitive pulp to the action of the many fluids and the various matters taken into the mouth. In this case the pulp itself will be more or less diseased. The exposed surface may be inflamed, and throw off a discharge, or it may have receded partially or wholly from the pulp cavity, leaving the vacated space to be occupied by extraneous matter. So that it is of considerable practical importance to learn whether the pulp has been exposed by the removal of softened dentine during the operation, or whether it has been previously exposed. In the one case, plugging, if so managed as not to produce pressure on the nerve, may be successful; in the other, it will, if performed, be followed by ill consequences, as the discharge from the pulp will be confined, and the usual distressing symptoms, indicating pus confined in an unyielding cavity, will result. It is a very good practical rule, if, in removing the softened dentine, or in pressing a probe in the cavity, pain is felt, but only so long as the instrument remains in contact with the tooth, to proceed to plug the cavity; but, if the pain continues after withdrawing the instrument, to postpone plugging, and resort to some means to restore the pulp to a healthy condition, or to produce its destruction; for the continuance of the pain is a tolerably sure sign that the pulp is more or less involved in disease, and that if the tooth be immediately plugged that inflammation will supervene.

The diseases of the pulp, and their treatment, as also the operation of plugging, will occupy our attention on a future occasion.

### LECTURE XI.

**DISEASE OF THE DENTAL TISSUES—NECROSIS—EXOSTOSIS—ABSCESS IN THE SUBSTANCE OF THE DENTINE—LOSS OF ENAMEL AND DENTINE FROM THE ANTERIOR SURFACE OF THE TEETH—ABSORPTION OF THE FANGS OF THE PERMANENT TEETH—CRACKS IN THE ENAMEL—PAIN IN SOUND TEETH.**

Dental necrosis, by which is meant the death of the whole or a part of a tooth, but, unlike caries, is not usually attended by decomposition of the dead tissue. When the whole of a tooth loses its vitality we have complete necrosis; when only part dies we have partial necrosis. In complete necrosis the fangs are entirely denuded of living periosteum, and the pulp dies; in partial necrosis the dead portion of the fang only is deprived of its periosteum, and the pulp of the fang in the majority, though not in all cases, dies.

The physical changes in the dental tissues indicative of complete necrosis commence with the death of the tooth, but from their slow progress some time elapses before they become discernible. The tooth gradually assumes a darker hue than natural, which increases in intensity till it is almost black. The dental periosteum gradually detaches itself from the fang, the tooth becomes loose, and, unless held in by the crooked form of the roots, drop out. The surface of the fangs are generally rough, and, near the neck, dotted over with nodules of hard green-colored tartar, while the ends of the roots often look worm-eaten, as though absorption had commenced. In some cases, where the teeth are dark and look wholly dead, and produce irritation in the alveolus, we find, on some parts of the fang, patches of newly-formed cementum, to which the periosteum is strongly attached, and the tooth is thus firmly held in its place. In these instances the death of the tooth must have proceeded gradually, and the periosteum subjected to that species of irritation leads to the formation of cementum. Dental necrosis may result from mechanical violence, or from inflammation of the alveoli; severe salivation and fever are sometimes productive of this disease; absorption of the alveoli with advancing age may also lead to necrosis; but usually, the tooth in old age is connected with the dental periosteum, and the pulp lives till the tooth, having lost all natural support, is forced out.

The effects of death of a tooth upon the neighboring parts vary with the nature of the producing cause; if a severe blow be the cause, the inflammation consequent upon the injury done to the tooth and alveoli will be acute, and if not wholly caused, in the first instance, by the death of the tooth, but arises from the injury the parts have conjointly received, will be kept up and even extend so long as the tooth is allowed to remain. But if, on the other hand, the dental necrosis be the consequence of a slight blow, or result from any deranged state of the system, the gum and alveoli may become but slightly inflamed and thickened; pus will be discharged from the alveolus, and cease only with the removal of the tooth; whether this be effected by extraction or by absorption of the alveoli and consequent extrusion of the dead tooth.

Mr. Fox in his practice attempted to save teeth where the pulp was exposed and painful, by carefully dislodging the affected teeth from the alveoli, and thus breaking off their connection with the nervous system. In some cases the experiment was successful, the tooth reunited to the periosteum of the alveolus, but in the great majority of cases it did not reunite, and the tooth then, of course became dead. In narrating these cases he gives an excellent description of the effects of sudden and complete necrosis of a tooth upon the alveolar periosteum. He says, "the method I adopted was to raise the tooth, by the common operation of extraction, so high in the socket as carefully to break the nerves and vessels which enter the extremity of the fangs: then, in withdrawing the force, to put the tooth back again into its former situation. I not only recommended this operation, but performed it upon a great number of persons, and for a short time, flattered myself with very sanguine expectations; these, however, were gradually destroyed, for some of my patients, in about three or four weeks, came complaining of pain, and were anxious to have the tooth completely removed. They did not suffer the tooth-ache so acutely as before, but the tooth had become loose and was protruded from the socket, so that whenever the mouth was closed the pressure of the teeth of the other gum against the tender one occasioned great pain. On extracting these teeth I found the fangs covered with a considerable quantity of coagulated lymph." I have seen many cases, where teeth, dislocated from a fall, have been returned to the alveoli, but could not be retained; if they are speedily removed we find a coating of what Mr. Fox calls coagulated lymph, but if allowed to remain a little longer, the lymph is replaced by, or infiltrated with pus.

The consequences upon the neighboring parts, when necrosis occurs without previous absorption of the alveoli, and the tooth is allowed to remain, are often more serious than those I have at present noticed. The periosteum of the alveoli becomes inflamed together with the neighboring parts: if the case be still neglected, the adjoining teeth are not unfrequently lost, and necrosis of a considerable part of the jaw may also result, Cases of this serious description sometimes come from a blow, but occasionally result from alveolar inflammation. Should, however, the inflammatory action be less severe, the gum and alveolus of the dead tooth may alone be affected; the tooth will then become gradually loose, the gum will be inflamed and thickened, and pus will be discharged from between the tooth and alveolus whenever the gum is pressed: this continuing, the tooth becomes more loose, and at last falls out, and the gum resumes a state of health. In teeth where necrosis has been gradually produced the tooth is generally wholly deprived of periosteum: when the death has been the result of violence, or of active alveolar inflammation, you will, on removing the tooth, find shreds of dead periosteum, infiltrated with pus, adherent to the surface of the fang. In partial necrosis of teeth the extent of the disease may be very limited, or the greater portion of the tooth may be involved; but, whether the extent be great or small, the necrosed portion is deprived of periosteum, and presents, on examination, a more or less rough surface. Whether or no the dentine be discolored will depend upon the situation of the necrosed portion. Thus, instances are not uncommon when the pulp of the tooth has died while the external surface of the fang has preserved its vitality. In these cases the dentine becomes discolored, and gives a general dark appearance to the tooth. On the other hand, nothing is more common than necrosis of the extremity of the fang while the remaining portion of the tooth retains its vitality; but here we find no discolorment, though the extent of the necrosis is clearly marked by the adhesion of the periosteum to the living portion, and its separation from the dead portion of the fang. In all cases of partial necrosis where the neck of the tooth retains its periosteal covering, with which I have become acquainted, the separated periosteum forms a sac round the dead dentine, the inner surface of which sac secretes pus, and, in fact, forms the coat of an abscess. Partial necrosis seems generally the result of inflammation of the dental periosteum or of the pulp, and may be situated in any part of the tooth, but it is far more common to find it about the apex of the fang, resulting from inflammation of the periosteum communicated by inflammation of the pulp. But instances are not very rare in which the necrosed portion occupies a circumscribed spot on the fang, or below the fang, of a molar tooth. The following is generally typical of such cases. A male patient of twenty-eight, applied to have a tooth removed, in consequence of severe pain. On examination, the tooth, a first molar of the upper jaw, appeared perfectly sound and healthy, but the gum was inflamed opposite the root of the tooth. The gum was lanced, a little pus escaped, and the pain ceased. In a short time the patient again applied, for relief from severe pain in the same situation, and insisted on the tooth being removed. He said that excessively severe pain had, at short intervals, attacked this tooth, and though, generally, reduced by lancing the gums, yet the inconvenience was too great to be endured for the sake of the tooth. On removal, a circumscribed spot of necrosed dentine was found between the fangs, from which the periosteum had separated and dilated, thus producing absorption of the alveolus between the fangs. The so-formed sac was full of pus: the tooth was otherwise healthy, and of good color. Whether inflammation of the periosteum, and consequent separation, was the primary condition, or whether necrosis was the first abnormal state, would be difficult to determine, but that the necrosed condition of the dentine was the cause which kept up the diseased state of the periosteum admits of no doubt.

Towards the middle or later period of life, the molar teeth are occasionally rendered painful and useless from necrosis of one of the fangs. I will recite a case that recently occurred to me. A male patient, aged 48, applied to be relieved of a troublesome tooth, of which he gave the following history. Twelve months since the tooth became slightly uneasy on contact with hot or cold fluids; after a while he discovered that it was slightly loose; still mastication produced little or no pain. The inconvenience gradually increased, till at last severe pain was felt whenever fluid differing in temperature from the mouth came in contact with the tooth. Pain, too, was occasionally produced by mastication. On examining the tooth—a second molar of the upper jaw—about the crown no morbid appearance presented itself, but the inner fang seemed more exposed at its base than usual. The gum about it was a little thickened, but not inflamed; a probe, however, could readily be passed down into the socket without meeting any obstruction: this at once proved the fang to be necrosed. The tooth was removed, and the inner fang was removed, and the inner fang was found to be perfectly unattached, was rough and discolored. The two external fangs were healthy, and the tooth in no part carious. Had this tooth been allowed to remain, the process of absorption of the alveolus and gum of the affected fang, which had already commenced, would have gradually advanced, and the fang in the process of time would have been left exposed in the mouth. The case I have detailed to you is not in any way peculiar; it is, in fact, a specimen of a very common complaint, the only remedy for which is extraction of the tooth. In these cases the pain felt on the application of hot or cold fluids is, I believe, seated in the alveolus occupied by the dead fang, which, being unattached, not only prevents the alveolus from filling up, but allows foreign matter to get into it, and keep up irritation, as well as producing irritation itself. Distinguished from total necrosis of a fang is death and discoloration of one side only: we have occasional opportunities of seeing in the molars of the upper jaw, the lingual side, or the buccal side of the gum and alveolus, completely absorbed, leaving the fangs completely exposed in their whole length, while those surfaces of the fangs which are opposed to each other are strongly attached to the periosteum and to the alveolus, which lies between the fangs. In these cases but little uneasiness is felt; the teeth remain useful to the last.

There is yet another form of partial dental necrosis: the pulp sometimes dies, and with it the surface of the pulp cavity, while the outer surface of the fang retains its vitality. The tooth, in this case, gradually darkens in color, but the periosteum remaining healthy, the tooth is retained, and may be useful for years. On the other hand, the periosteum may be slightly affected and yet practically give no inconvenience to the patient. Such teeth are usually retained, and continue useful for an indefinite period. But, unfortunately, the periosteum of alveolus, and of the tooth, continuing in this state, is rendered very liable to inflammation, which, on its occurrence, renders the removal of the tooth imperative. In the operation of pivoting, or grafting, as it is sometimes called, the crown is removed, while the fang is permitted to remain in the gum. The pulp of the fang is destroyed by a fine steel probe, and the cavity enlarged to hold a piece of gold wire, to which is attached the crown of a foreign tooth. Here we produce death of the pulp, and hence necrosis of the inner portion of the fang. The external surface of the fang may, and does, in the majority of cases, remain healthy, though in a few individuals, unfortunately, the opposite result occurs. The fang becomes necrosed, the periosteum inflames, and the fang would be eventually dislodged, but that the degree of risk of more extensive mischief and of suffering, from the inflammation of the surrounding textures, renders the removal of the fang necessary long before nature could have completed her process of extrusion.

I shall describe several cases in which serious consequences followed the operation of pivoting, when we come to the consideration of that subject.

*Dental Exostosis, or Hypertrophy of the Cementum of the Fang.*—It will not be forgotten that the surfaces of the fangs of teeth are coated with a thin layer of cementum. Under certain circumstances this layer becomes increased in thickness by additions on the external surface. The newly added cementum is in every way similar in structure to that previously forming part of the tooth.

You have learned from your pathological lecturer, that a natural tissue increased beyond the natural amount constitutes a disease, by changing the size and figure of the affected organ, and hence encroaching upon neighboring parts. When the cementum of the tooth becomes developed in an unnatural degree, the form of the tooth becomes changed, and we have exostosis. With the increase of size in the fang of the tooth there must be a corresponding increase of capacity in the containg alveolus. In dental exostosis the amount of new cementum may be very slight, or it may be considerable in quantity. The affected fang may be but slightly enlarged, or it may be increased to twice its natural size. Near or about the end of the fang is the most common situation to find the greatest amount of cementum: but you seldom find an increase on one side of the fang only, unless the opposite side has been exposed by the absorption of the gum, or deprived of its periosteal covering. It is not rare to find the thickening commence abruptly above the necrosed extremity of the fang usually present in chronic gum-boil. You will also find cases in which the dentine of a fang has been necrosed, and the fang itself been thrown up and lying in its length on the surface of the gum, held in that situation by a patch of new cementum, which has been deposited on and is adherent to the stump. The periosteum of the new cementum then connects the stump with the gum. A short time since, a patient applied to have the fang of a second bicuspis removed. She stated that she had suffered a great deal of pain, which, commencing in the tooth, extended to the side of the face and the head. The pain was remittent, lasting at one time for many hours, at another time only for a few minutes. It was not until after several attempts had been made, and with the application of considerable force, that the removal of the offender was effected. There was great exostosis, and the difficulty arose from the fang near its extremity being twice the diameter of the neck, where it was closely embraced by the alveolus. The surface of the hypertrophied part, like that of other teeth similarly affected, was smooth, but pitted, unlike the smooth and even surface of the fang of a healthy tooth. The orifice admitting the vessels and nerves into the pulp cavity, small in the healthy tooth, is comparatively large in the new cementum of exostosis, and its edges are rounded. Dental exostosis is caused by that condition of the periosteum which is called irritation—a state usually induced by pre-existing disease in other dental tissues, and, in a great majority of cases, by caries: not always, however. In the specimen before you, from which the figure has been taken, the tooth appears, in all respects, healthy, excepting the exostosis of the posterior fang. In bones we find exostosis situated in the immediate neighborhood of inflamed parts. It is also exceedingly common about the sheaths of the tendons of the fore-legs of horses subject to be rode at a fast pace on hard roads, whereby the feet and legs become injured, and often slightly inflamed, and is called a *splent.*

The fangs of teeth that have been pivoted are very liable to exostosis, and with it thickening and increased vascularity of the gum belonging to the tooth. In teeth connected with chronic gum-boil from necrosis of the extreme end of the fang, exostosis of the upper part of the fang is very common.

In all these instances we find hypertrophy of the cementum brought about in or by the vicinity of diseased action.

The *symptoms* of dental exostosis are not in all cases sufficiently distinct from those indicating other diseases to enable the surgeon to recognise the exact nature of the malady. When fangs that have lost their crown by caries are subject to this disease, the gums are usually thickened, are of a deep dull red color, and bleed readily. The surface of the gum is sometimes smooth and shining; at others is rough and granulated, and the edge, instead of becoming gradually thinner to its termination on the neck of the tooth, ends by a thick, broad, flat edge, in which change of form the edge of the alveolus also participates. The condition of the gums I have just described is very commonly seen when the incisores of the upper jaw are affected with exostosis, and I have observed is frequently confined to the anterior or labial surfaces, while the surface of the gums within the mouth are comparatively free from disease. We may, however, have exostosis without any disease in the gums. Pain is not a constant attendant on exostosis, though in most cases it is occasionally present, and when present, is generally of a gnawing character. In cases of sympathetic affections induced by dental exostosis, pain in the affected teeth, so far as my experience goes, is very commonly absent.

Of the various dental maladies, exostosis is the most frequent source of sympathetic disorders. The affections so induced are usually confined to functional derangement of the whole nervous system, or the nerves of some particular part.

I need not do more than remind you that, in whatever part of its course a nervous fibre be subject to irritation, the effect produced by that irritation, whether it be suspension or derangement of function, will be felt in the part to which the nerve is ultimately distributed, and that you may or may not have pain in the part where the irritation is applied. Again, if the whole or part of the nervous system be predisposed to any special disease, irritation applied to any set of nerves may induce that disease, the degree of irritation being wholly insufficient to excite the sympathetic malady had there been no predisposition. Thus, diseases of the teeth will occasionally produce epilepsy where there is a predisposition to that complaint. Tic douloureux is also sometimes brought on by the like cause. The following case will illustrate the point under consideration: A lad, a farm laborer, from Windsor, was admitted into the hospital for epilepsy. The usual remedies were tried for six weeks without effect. His mouth was then examined, and the molar teeth of the lower jaw were found to be much decayed, and of some of these the fangs only remained. He did not complain of pain in the diseased teeth, or in the jaw. The decayed teeth were, however, removed, and the fangs of each were found to be enlarged and bulbous from exostosis. At the expiration of eighteen months he had not had a single fit since the removal of the diseased teeth, though for many weeks previous to the operation he had two or three per day. This is a case of singular interest, inasmuch as there was no complication of maladies, and hence there could be no doubt as to the cause of the disease, seeing that it immediately subsided when the teeth were removed; and it is further useful, in showing that a sufficient source of local irritation to induce functional derangement may exist without pain being felt in the part where the irritation is applied.

A similar but less marked case occurred shortly afterwards in the person of a policeman. He had fits, which were greatly relieved by the removal of the inferior wisdom tooth, the subject of exostosis.

Vague and shifting pains about the head and face, and sometimes even in the neck and shoulders, are not uncommon consequences of dental exostosis. Tic douloureux is also sometimes induced by the like cause, though much less frequently. I have seen tooth after tooth removed for the relief of tic, where no benefit has followed; but, on the other hand, several cases have come to my knowledge where the pain gradually diminished from the time the teeth were extracted, and shortly wholly subsided. I should here remark, that, when pain has become periodical, and has existed for some time, the attacks will not necessarily cease immediately, the cause which originally produced them has been removed; on the contrary, they frequently appear for some little time afterwards, but in each successive attack the pain diminishes in intensity, and at last ceases. I have observed that the removal of a tooth which has occasioned sympathetic pain almost always produces a severe paroxysm of the sympathetic pain, and that the paroxysm will bear some relation in duration and intensity to the previous attacks. In accounting for the production of these sympathetic maladies, I must beg you for a moment to bear in mind, that a sympathetic affection in one part is a consequence of organic or functional derangement in some other part of the body, but for which the sympathy would not exist. The most familiar example illustrative of the point is, perhaps, the pain felt in the shoulders when the functions of the liver are deranged. It is not in all cases very easy to see why, when we have diseases in one part, we should also have pain in another, the parts being apparently disconnected, and at some distance from each other; and it is still more difficult to see why the sympathetic pain is not constant in all similar cases of the primary affection. Anatomical examination will, however, enable us to understand how many sympathetic affections may arise from dental disease.

Now you are well aware that some tissues, and especially bone, are absorbed, and make way for a growing tumor, and that the process is pretty generally unattended with pain. Not so, however, with the larger trunks of nerves: they, instead of being absorbed, are pressed before a growing tumor, become strained, and hence there is pain or suspension of the nerve's function, either in the part itself, or in those parts to which the nerve is ultimately distributed. The trunks of the dental nerves contain many fibres that are to be distributed to other parts than the teeth.

Bearing, then, these facts in mind, we can at once understand that, if the extremities of the fangs of a tooth be but slightly increased in size, whether by hypertrophy of the cementum or by the growth of any other tumor, the dental nerve may be thereby disturbed, and hence sympathetic pains may be induced in any of those parts with which the nerve is connected. These interesting physiological points will, however, be brought more fully under your notice in other lectures than those on dental surgery.

### LECTURE XII.

DISEASE OF THE DENTAL PULP—IRRITATION OF THE DENTAL PULP—CAUSES AND TREATMENT—INFLAMMATION, GENERAL AND PARTIAL, OF THE PULP—CAUSES AND TREATMENT.

### *Diseases of the Dental Pulp.*

THE dental pulp, composed of vessels, nerves, and the remains of the formative tissue, is, like other vascular textures, liable to irritation, to inflammation, and its events. These, in their various forms and consequences, it will now be our business to trace.

*Irritability of the Pulp.*—By irritability here is meant, an increased susceptibility to pain and to morbid action, unattended with organic change. A cause productive of no inconvenience in a healthy tissue will in an irritable one induce pain and increased action, and, if long continued, inflammation, and its various results. The most frequent cause of irritability is caries, immediately prior to its laying open the cavity. When a tooth is in this state, the pulp, having its natural defence much weakened, is, consequently, much exposed to sudden changes of temperature, and also to the contact of the fluids of the mouth, by their oozing through the diseased or disorganised dentine. Hence the pulp becomes irritable; so that when hot or cold, or sweet or acid matters are taken into the mouth, and come upon the tooth, a more or less severe twinge of pain is felt, which may last but for a moment, or may continue for some minutes, and then subside, the time varying in each case with the degree of irritability of the pulp. Again, if the pulp or periosteal membrane of a tooth be inflamed, the pulps of the neighboring teeth may become highly sensitive, and even painful, and, consequently, unfitted for use. Sometimes pain in one tooth will be followed by pain in the corresponding tooth of the opposite side, and even of the opposite jaw; indeed, occasionally disease of a pulp itself, unattended with pain, will induce pain in the pulp of another perfectly sound tooth. In these cases we can explain the irritation produced in neighboring teeth only on the supposition that the nervous fibrils destined to be distributed to the affected tooth are irritated in their passage past the pulp that is really diseased. Irritation of the dental pulp terminates in one of three ways:

*First*, the cause being removed, the irritation subsides, and the pulp returns to health, as instanced where a tooth with an inflamed pulp has been extracted, other teeth that have been affected by it become again healthy. Again, a tooth irritable from incipient caries becomes healthy after the cavity has been plugged.

*Secondly*, the pulp may become converted into dentine opposite the point from whence the source of irritation acted, and thus form a barrier for itself against the ingress of the irritating agent. We see instances of this event of irritation in cases where the pulp is converted into dentine opposite a point from which a portion of the enamel and of the dentine composing the crown has been removed by wear, by fracture, by the use of the file, or even by caries.

Indeed, if the pulp of a tooth extracted for caries and subsequent odontalgia be carefully examined, there will, with a few exceptions, be found more or less calcification near the point towards which the disease had advanced.

*Thirdly*, the irritation may terminate in inflammation and suppuration of the pulp. This result usually follows long-continued irritation, arising from a local cause, as caries, or simple fracture of a tooth.

*Treatment.*—Where one tooth is rendered painful in consequence of disease in another tooth—that is, is sympathetically affected—the treatment must be addressed to the primary offender, the cure or removal of which will be followed by the subsidence of the irritation of the one sympathising. Should, however, the irritation have a local origin, and arise from loss of substance in the crown of the tooth by caries, the tooth should, without delay, be carefully plugged with gold or other stopping; the irritating agents will then be hindered from filtering through upon the pulp; and though the metal used for plugging transmits changes of temperature more readily than the sound portion of the tooth, yet the pulp soon becomes inured to this source of irritation, or shuts it out, by calcifying opposite to the plug—an event very desirable in all such cases. A sudden change of temperature, especially from hot to cold, will, in most teeth that have been recently plugged, supposing the plug to be of any size, produce sharp pain for the moment. The paroxysm of pain, however, quickly subsides, and, after a while, cannot be reproduced by a similar cause.

If the loss of substance in the crown arises from fracture, time, with the application of astringents to the exposed and sensitive dentine, together with a careful avoidance of unnecessary sources of irritation, will effect the cure. If the crown has suffered from undue wear, similar treatment should be pursued, with a more careful use of the teeth. If the bicuspides or molares are reduced to this state, a cap of gold may be fitted to them, on which the patient may masticate for a time. Should this, which is often a most serviceable plan, be adopted, the cap must be removed several times in the course of the day, and the tooth or teeth carefully brushed: it must also be left off during the night, otherwise the teeth may become the subjects of caries.

*Inflammation of the Dental Pulp.*—The pulp, in common with all vascular tissues, is liable to inflammation, and, like those which are confined in an unyielding case, is the seat when inflamed of violent throbbing pain. We are most of us pretty well acquainted with the sensations which accompany inflammation of the dental pulp, seeing that that condition is, in nine cases out of ten, the cause of tooth-ache.

The structural and physical changes of texture that accompany or constitute inflammation are very much the same, whatever soft tissue may be the seat of the disease. I need not, therefore, occupy your time in a minute description of these, but shall content myself with noticing those appearances that generally accompany inflammation of the dental pulp. The possibility of swelling is precluded by the dense case in which the pulp is contained, unless this is somewhere imperfect, and then the enlargement can only occur at that point. Redness is, however, always present, and is occasioned by an increased size of the capillaries; these vessels, invisible to the naked eye when healthy, become very apparent under inflammation. The healthy pulp is a very light pinkish grey, but an inflamed pulp is bright scarlet. The best method of getting at the pulp for examination is to screw the body of the tooth in a vice till it breaks. The broken pieces may then be carefully removed, and the pulp examined. This plan is preferable to sawing through the tooth, for then the saw-dust gets upon the diseased part, and obscures the view.

The whole of the pulp may be involved in disease, or only a portion, the amount chiefly depending upon the nature of the producing cause, or, in other words, there may be *partial* or *general* inflammation of the pulp. Thus, if the pulp cavity be entire, and the pulp be subjected to prolonged irritation, the whole substance of the organ will become inflamed; on the other hand, if an opening has, from any cause, been made into the pulp cavity, the disease may be confined to that part situated near the opening. It is not difficult to conceive why this should be, when we consider that in the latter case the surface subjected to the direct action of the irritating agent will be confined to that exposed by the opening; and, further, that any discharge poured out from the inflamed part will find a ready passage for escape, and hence produce no direct influence upon the unaffected part of the pulp, whereas, had the pulp cavity been entire, any swelling of or discharge from one part of the pulp finding no escape, would press equally on all parts, and thus the whole organ would become involved in disease. Again, the inflammation may be general and acute, and speedily terminate in the destruction of the pulp, or, on the other hand, it may be chronic, and the pulp may, under disease, retain its vitality for a considerable time. The degree of the inflammation will depend upon the character of the producing cause, upon the previous state of the pulp and of the walls of the pulp cavity, and also upon the state of the system at the time of the attack. If the pulp has been rendered irritable by gradual thinning of the walls of the cavity, the inflammatory action will probably be general, and terminate in destruction of the part, as illustrated in the following case.

A short time since, a patient applied for treatment who stated that she had suffered severe throbbing pain in a first molar of the lower jaw for two days, that the pain, at first slight, had gradually increased in intensity, and that the adjoining teeth had now become tender, so that the mouth could not be closed without pain. The aching tooth had suffered from superficial caries of the masticating surface, which disease had thinned the walls, but had not made an opening into the pulp cavity. After removal, the tooth was very carefully examined, and the cavity found to be perfectly entire. The pulp had been inflamed, was dead, offensive, and bathed in pus, as ascertained by the microscope, while in its substance a few small nodules of dentine could be distinguished. The periosteum of the tooth was becoming slightly affected about the extreme point of the fang. This case presents no peculiarities, but is on the contrary typical of general and acute inflammation and subsequent suppuration and death of the dental pulp, arising from prolonged irritation, caused either by irritating fluid oozing through the thinned wall of the pulp cavity, or by sudden alternations of temperature arising from the same cause. A similar state of disease not unfrequently comes on from exposure of the pulp by caries; in such cases the inflammation commences at the exposed point, and extends to the rest of the pulp. Another cause not unfrequent among the more wealthy, is the closure by plugging of a carious opening through which a small portion of discharge thrown off from the exposed portion of the pulp had habitually escaped. Compression of the pulp by the bending or bulging inwards of the softened or thinned wall of the cavity, or mechanical injury from any cause, is also an occasional excitant of general inflammation. The pain attendant upon general inflammation of the pulp mostly commences with slight gnawing, which gradually increases and becomes throbbing, and lasts till the pulp is destroyed. The pain then ceases or is altered in character to a dull heavy pain, and at this period the side of the face very commonly becomes more or less swelled and œdematous. This change of symptoms indicates that the pulp has been destroyed, and the occurrence of dull heavy pain, that the periosteum has become the seat of inflammation. Supposing, however, the inflammation to have resulted from compression, then the pain will of course set in suddenly. If, again, the general is the consequence of previous local inflammation, as the one is a mere extension of the other, the exact period of change cannot be declared. General inflammation, when there is no outlet from the cavity, excepting through the fangs, unless arrested at an early stage, terminates in suppuration and death of the pulp. If there be an opening into the cavity the inflammation may, perhaps, partly subside, but the pulp seems never to return to a state of health. Again, if the inflammation result from exposure from fracture of the crown of the tooth, destruction of the pulp is the usual consequence.

The consequences of general inflammation of the dental pulp that may be entailed on the neighboring parts are numerous and varied. The violent pain may cease and the face swell, the swelling may subside, and the tooth lose all tenderness: the patient soon forgets the attack, but if the tooth be examined it will be found to have assumed a greyish hue, thereby indicating that the dentine of the pulp cavity is necrosed, and that the tooth holds its vitality from the periosteum alone. The tooth, however, may remain useful for years; hence, this is the most favorable result of general inflammation of the pulp; but unfortunately it is a rare one. It is far more common for the inflammatory action to extend to the periosteum of the fang, and thus induce death of the end of the fang and consequent gum-boil. Indeed, the disease may not stop here; the maxillary periosteum may become inflamed, and lead to necrosis of a large part of the jaw. Two cases in which this has occurred are now in attendance at the hospital.

*Treatment.*—Inasmuch as the pulp very rarely becomes the subject of general inflammation unless the crown has been previously injured, either by caries, wear, or mechanical violence, it will be better to remove the tooth at once, rather than to endure the pain that would be consequent on its continuance, and run the risk of injury to the neighboring parts; unless, indeed, it be a front tooth of the upper jaw, where, in some patients, it is desirable to preserve the fang for pivoting; in that case the crown only must be cut off and the pulp destroyed. If the disease has resulted from plugging, the plug should be immediately removed, by which measure if the pulp itself is not saved, yet the disease will possibly not extend to the periosteum of the tooth. Several years since I had a tooth plugged; the cavity was occasioned by caries, and the pulp was exposed at one point, but whether before or during the operation I could not tell. I experienced considerable pain at the time, but within an hour the tooth became quite easy; for two months I had no pain or uneasiness, but at the end of this time a gnawing pain commenced, which gradually increased and became throbbing. At the end of twelve hours from the commencement of the pain I removed the plug; the pain ceased within a few minutes, and the mouth was filled with a most disagreeable fetid taste. On subsequent examinaticn it was found that the body of the pulp had perished, while that occupying the fang had survived.

I have found in practice that it is far from uncommon for a tooth plugged after the pulp has been slightly exposed, but previous to pain being suffered, to answer for one, two, or even three months, and then to be attacked with inflammation of the pulp; thus obliging the removal of the plug or the extraction of the tooth. In practice, therefore, whenever you find a plugged tooth attacked with gradually increasing pain, the plug should be immediately removed; then, if the pain ceases and the tooth be of any service, it may be retained. In such case the cavity should be filled daily with cotton wool, saturated in a solution of mastic in spirits of wine; this makes a good temporary plug for the exclusion of food, and may be used till the tooth will bear a more permanent stopping.

